# Influence of Novel Highly Pathogenic Avian Influenza A (H5N1) Virus Infection on Migrating Whooper Swans Fecal Microbiota

**DOI:** 10.3389/fcimb.2018.00046

**Published:** 2018-02-22

**Authors:** Na Zhao, Supen Wang, Hongyi Li, Shelan Liu, Meng Li, Jing Luo, Wen Su, Hongxuan He

**Affiliations:** ^1^National Research Center for Wildlife-Borne Diseases, Institute of Zoology, Chinese Academy of Sciences, Beijing, China; ^2^Key Laboratory of Pathogenic Microbiology and Immunology, Institute of Microbiology, Chinese Academy of Sciences, Beijing, China; ^3^College of Environmental and Resource Sciences, Zhejiang University, Hangzhou, China; ^4^Department of Infectious Diseases, Zhejiang Provincial Centre for Disease Control and Prevention, Hangzhou, China

**Keywords:** migrating birds, whooper swans, fecal microbiota, highly pathogenic avian influenza A virus, H5N1

## Abstract

The migration of wild birds plays an important role in the transmission and spread of H5 highly pathogenic avian influenza (HPAI) virus, posing a severe risk to animal and human health. Substantial evidence suggests that altered gut microbial community is implicated in the infection of respiratory influenza virus. However, the influence of H5N1 infection in gut microbiota of migratory birds remains unknown. In January 2015, a novel recombinant H5N1 virus emerged and killed about 100 migratory birds, mainly including whooper swans in Sanmenxia Reservoir Area of China. Here, we describe the first fecal microbiome diversity study of H5N1-infected migratory birds. By investigating the influence of H5N1 infection on fecal bacterial communities in infected and uninfected individuals, we found that H5N1 infection shaped the gut microbiota composition by a difference in the dominance of some genera, such as *Aeromonas* and *Lactobacillus*. We also found a decreased α diversity and increased β diversity in infectious individuals. Our results highlight that increases in changes in pathogen-containing gut communities occur when individuals become infected with H5N1. Our study may provide the first evidence that there are statistical association among H5N1 presence and fecal microbiota compositional shifts, and properties of the fecal microbiota may serve as the risk of gut-linked disease in migrates with H5N1 and further aggravate the disease transmission.

## Introduction

The H5N1 subtype of highly pathogenic avian influenza virus (HPAIV) continues to cause outbreaks in poultry and sporadic human infections, thus posing a persistent potential pandemic threat (WHO, 2016). Wild birds, especially waterfowl of the order *Anseriformes* (ducks, geese, and swans) are the natural reservoir of avian influenza viruses (Sturm-Ramirez et al., 2005; Olsen et al., 2006). However, apart from sporadic cases, widespread migratory birds have been the victims of HPAI since at least 2005 in Qinghai Lake, China (Liu et al., 2005). It is believed that overlapping migratory flyways help circulate H5 HPAIVs amongst different bird species, and allow the spread of the virus across continents (Sturm-Ramirez et al., 2005). The migration of wild birds plays an important role in the transmission and spread of H5 HPAIVs, posing a severe risk to animal and human health. Whooper swan (*Cygnus cygnus*) is a large Northern Hemisphere swan which breeds in subarctic Eurasia. They are migratory wintering in northern Europe and eastern Asia, such as China, Korea, and Japan. And it is an occasional vagrant to western North America (BirdLife International, 2016). Whooper swan is considered to be a highly susceptible species to HPAIVs among wild birds. The reported outbreaks of HPAIV (H5N1) infections in whooper swan once occurred in China (Chen et al., 2006) and Mongolia in 2005, Iran, Germany (Teifke et al., 2007), France, Denmark, the United Kingdom, and Mongolia in 2006, Russia in 2007, and Japan in 2008 (Uchida et al., 2008).

In January of 2015, another novel recombinant H5N1 virus (Clade 2.3.2.1c) emerged and killed about 100 migratory birds, mainly including whooper swans and pochards in Sanmenxia Reservoir Area of China, the important wintering habitat when they migrated to China from Mongolia and Siberia (Bi et al., 2015). The novel H5N1 isolate is likely spread by the long-distance migration of wild birds, which might have been infected by the virus from domestic poultry during stopover sites in their migration. In this outbreak, it is noteworthy that other than severe lung damage, intestinal villus desquamation, lymphatic tissue cells neorobiosis and disaggregation under mucosa were also found in the small intestines of whooper swan who dead of the novel H5N1 virus (Bi et al., 2015). There is some evidence exhibit respiratory influenza infection induces gut immune injury by altering the composition of gut microbiota (Dilantika et al., 2010; Ichinohe et al., 2011; Wang et al., 2014; Qin et al., 2015; Deriu et al., 2016). Influenza H5N1 virus infection affect host microbiota in a mice model, however, the influence of H5N1 infection in migratory birds and the interplay between H5N1 infection and the fecal microbiota of the whooper swan remains unknown.

Additionally, our knowledge of the avian microbiota has arguably lagged behind that of many other vertebrates, most notably humans and mice (Waite and Taylor, 2014, 2015). Research foci in avian microbiota mainly included the bacterium variation along the GI tract, the impact of host age, dietary, probiotic manipulation and antibiotic treatment refer to Chicken, Turkey, Hoatzin, Kakapo, Kittiwake, Parrots, and Penguin (Waite and Taylor, 2014, 2015). While there is extensive evidence that gut microbiota can promote or reduce the severity of viral infection and progression, and on the contrary, viral infection can also impact the composition of gut microbiota in their hosts (Hooper et al., 2012; Moon and Stappenbeck, 2012; Wilks and Golovkina, 2012; Lynch, 2014). Not only the characteristic of fecal microbiota in migratory birds, but also the influence of IAV infection in gut microbiota still remain unclear. Considering the potential relationship between viral infection and the fecal microbiota (Lozupone et al., 2013; Moeller et al., 2013; Qin et al., 2014; Ling et al., 2015; Zhao et al., 2017), understanding how the composition of the microbiota changes as a result of H5N1 infection and the implications of these changes on the prevalence of avian influenza in migratory birds is vitally important.

To evaluate the role of H5N1 virus infection in shaping the fecal microbiota, to determine how H5N1 virus affects the content and stability of the fecal microbiota in whooper swan, and to understand the role and influence of H5N1 virus dynamics in this outbreak, we collected fecal samples from healthy individuals or individuals who were infected with H5N1 virus. We were thus able to investigate alteration of the fecal microbiota in individuals with or without H5N1 virus infection.

## Methods

### Biosafety and ethics statement

All experiments involving H5N1 viruses were performed in an Animal Biosafety Level 3 (BSL-3) containment laboratory in the Research Center for Wildlife Diseases, which was approved by the Chinese Academy of Sciences. This research was conducted according to the guidelines of the Good Experimental Practices adopted by the Institute of Zoology, Chinese Academy of Sciences and conformed to the regulatory requirements of Sanmenxia Reservoir Area, Henan Province, China. All experimental procedures and sample collection were approved by the Animal Ethics Committee at the Institute of Zoology, Chinese Academy of Sciences, China (Permit Number: IOZ10013).

### Sample collection

We describe migratory whooper swans with or without HPAI H5N1 virus infection in the Sanmenxia Reservoir Area of China, an important wintering habitat for wild birds migrating to China from Mongolia and Siberia. The H5N1-positive individuals are confirmed by the hemagglutination (HA) and hemagglutination inhibition (HI) assay with AIV antigens and antisera and viral RNA amplification and sequencing according to WHO standard protocols (WHO, 2011). In this outbreak, the adult individuals with clinical abnormality including weakness, respiratory difficulty, especially neurologic signs, such as tremors, seizures, severe incoordination, and torticollis, and the individuals suffer from an increased incidence of diarrhea in the absence of obvious enteric pathogens and increased intestinal inflammation were chosen. The feces from H5N1-positive or negative swans were collected for gut microbiota analyses.

A total of 20 fresh fecal samples from 20 swans (10 H5N1-positive individuals and 10 healthy controls, half male and half female in each group) were collected between January 9th and 12th, 2015. Fecal samples were collected immediately after defecation in the early morning. The fresh fecal samples were collected from the inside feces into sterile plastic container, snap-frozen in liquid N_2_, stored on dry ice, and delivered to the lab in 30-min, and immediately stored at −80°C until further analysis.

### DNA extraction from feces

The frozen aliquots (200 mg per aliquot) were added to a 2 ml screw-cap and thawed on ice until 1.4 ml ASL buffer from the QIAamp DNA Stool Mini Kit (Qiagen, Hilden, Germany) was added. The subsequent DNA extraction steps were conducted according to the QIAamp Kit protocol. The concentration of the DNA was measured using a NanoDrop™ 2000 (Thermo Scientific). Finally, the DNA was dissolved in 200 μl sterile ddH2O and stored at −20°C until use.

### Bacterial 16S rRNA gene amplification and sequencing analysis

The 16S rRNA genes were amplified in triplicate using primers (341F: 5′-CCTAYGGGRBGCASCAG-3′, 806R: 5′-GGACTACHVGGGTWTCTAAT-3′) specific for the V3-V4 region of the 16S rRNA genes. The PCR products were detected using 2% agarose gels, and samples with a bright strip between 400 and 450 bp were chosen. Then, the PCR products were purified using the Omega Gel&PCR Clean Up Kit (Omega Bio-Tek, USA). Sequencing libraries were generated using the TruSeq DNA PCR-Free Sample Preparation Kit (Illumina, USA) according to the manufacturer's recommendations, and index codes were added. The quality of the library was assessed using the Qubit® 2.0 Fluorometer (Thermo Scientific) and Agilent Bioanalyzer 2100 system. Finally, the library was sequenced on an Illumina Miseq platform, and 250 bp paired-end reads were generated.

### Bioinformatics analysis

Paired-end reads were assigned to samples based on their unique barcode and truncated by cutting off the barcode and primer sequence. The paired-end reads were merged using FLASH (V1.2.7, http://ccb.jhu.edu/software/FLASH/), and raw tags were successfully spliced. Quality filtering of the raw tags was performed to obtain high-quality clean tags according to the QIIME quality control process (V1.7.0, http://qiime.org/index.html).

Quality was controlled as follows: a. If the average quality score for a 50 bp sliding window was ≤19, then the tags were discarded from the original reads. b. Tags that contained more than 25% low-quality bases were removed.

Sequence analysis was performed with Uparse software (http://drive5.com/uparse/). Sequences with ≥97% similarity were assigned to the same OTUs. OTUs were initially rarefied at the depth of 36455. Low-level OTUs with abundance less than 1000 were filtered. A representative sequence for each OTU was screened for further annotation.

For each representative sequence, the GreenGene Database (http://greengenes.lbl.gov/Download/Sequence_Data/) was used to annotate the taxonomic information based on the RDP classifier (Version 2.2, http://sourceforge.net/projects/rdp-classifier/) algorithm.

### Statistical analysis

The analysis of alpha diversity, which included calculation of the observed species, Chao 1, ACE, Shannon, and Simpson index as well as the beta diversity analysis, which utilized both weighted and unweighted UniFrac, was conducted with QIIME (Version 1.7.0). Cluster analysis was preceded by principal coordinate analysis (PCoA) of Euclidean distances and Weight Unifrac among samples, and the results of all of the analyses were displayed in R software (Version 2.15.3) using packages “vegan,” “labdsv,” and “adehabitatHR.” To compare samples in two groups, Euclidean distances, Bray-Curtis dissimilarities and Weight Unifrac were calculated. Mann-Whitney U test was used to analysis whether distances and dissimilarities between H5N1-positive samples were higher than those among healthy controls. Additionally, Principal Component Analysis (PCA) was performed using log-transformed OTU matrix. In order to confirm the separation, K-means clustering was performed using R package “ClusterR” with K-means++ as initializer. Optimal number of clusters for k-means was determined using the average silhouette width of all clusters (the distance metric defaults to euclidean) as criterion and K-means++ as initializer.

## Results

### Characteristics of the individuals and 16S rRNA gene sequence data

We sequenced the V3-V4 region of the 16S rRNA genes in ten fecal samples collected from H5N1-infected individuals, in addition to five fecal samples from healthy controls in the Sanmenxia Reservoir Area of China on January 9th−12th, 2015. After quality trimming and chimera detection, an average of 51,387 high-quality sequences (ranging from 42,480 to 63,318) remained. After sample rarefaction, these sequences were clustered into operational taxonomic units (OTUs) at a threshold of 97% sequence identity.

**Figure 1:**
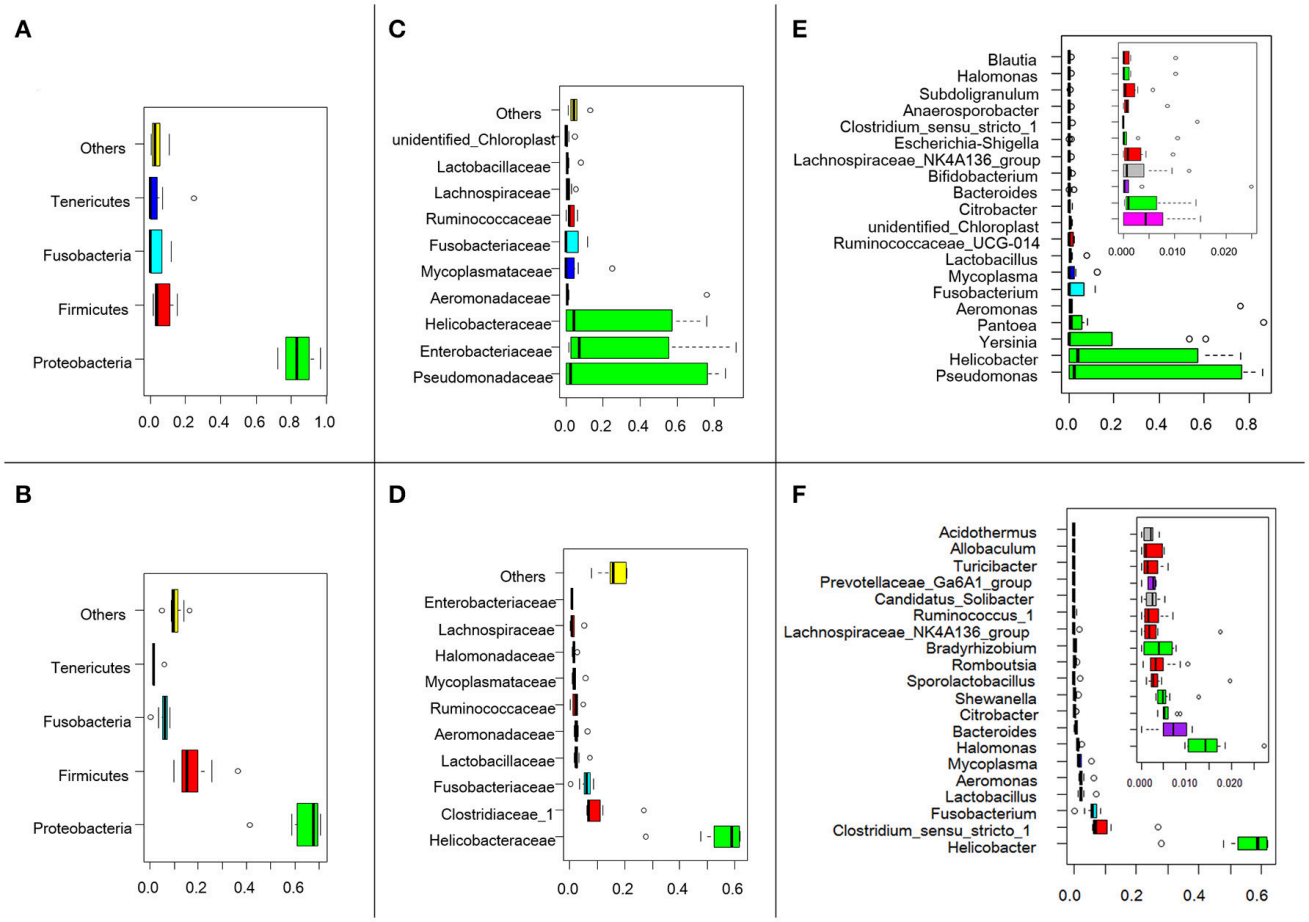
Swan fecal microbiota abundances and phylogenetic profiles at the phylum, family and genus levels for H5N1 virus infected individuals and healthy controls. A phylum abundance variation box plot for the top 4 phylum in the H5N1-positive samples **(A)** or healthy controls **(B)** is shown. The central rectangles represent the interquartile range (IQR), which spans the first quartile to the third quartile. The line inside the rectangle shows the median and “whiskers” above and below the box show the locations of the minimum and maximum within 1.5 IQR from the first and third quartiles. The circles represent outliers beyond the whiskers. The 10 most abundance family in the H5N1-positive samples **(C)** or healthy controls **(D)** is shown. And the 20 most abundance genera in the H5N1-positive samples **(E)** or healthy controls **(F)** is shown. The color of each family and genera corresponds with the color of its respective phylum.

**Figure 2:**
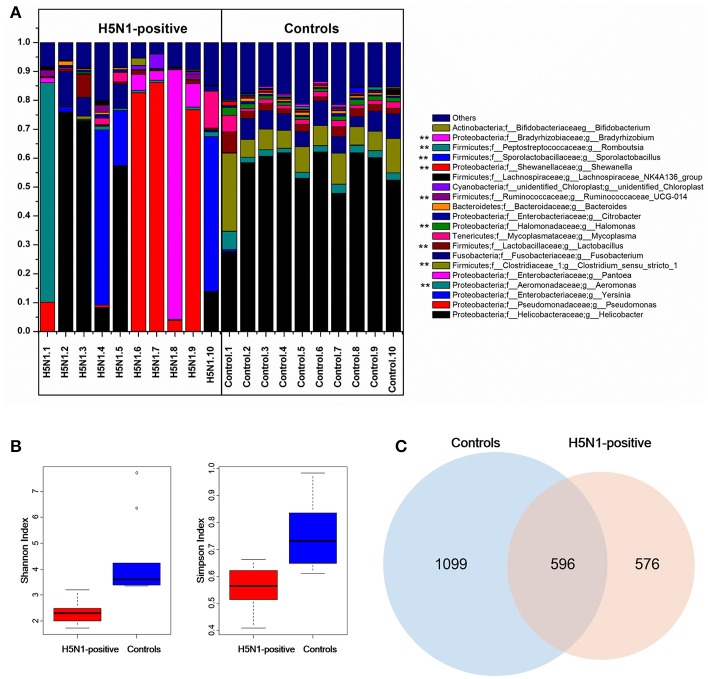
Comparison of the composition of fecal microbiota between H5N1-infected whooper swans and healthy controls. **(A)** Relative contribution of the top 20 dominant genera in each sample. Genera that differed significantly between the H5N1-infected individuals and the healthy controls are marked with stars (^**^p < 0.01 using Mann-Whitney *U*-test). The Shannon and Simpson **(B)** indices were used to estimate diversity (data shown as the mean ± SEM). Venn diagram illustrating overlap of OTUs in fecal microbiota between the individuals with or without H5N1 infection **(C)**.

The effective tags from the sequencing data were aligned against the reference sequences from database. The relative abundance of phylum, family and genus among H5N1-positive groups and healthy controls are shown in Figure 1. At the phylum level, *Proteobacteria, Firmicutes, Fusobacteria* and *Tenericutes* were dominant in the fecal microbial communities of all groups (Figures 1A,D). Compared with healthy group, H5N1-positive samples had fewer *Proteobacteria* and *Firmicutes* (*p* < 0.001, Mann-Whitney *U*-test). At the family level (Figures 1B,E), H5N1-positive samples had more *Enterobacteriaceae*, and fewer *Helicobacteraceae* and *Lactobacillaceae* (*p* < 0.01, Mann-Whitney *U*-test). At the genus level, *Helicobacter* and *Pseudomonas* were relatively abundant in most of the samples (Figures 1C,F). Each of the 20 predominant genera showed wide variation in abundance across the collected samples (Figure 2A). In particular, the abundance of *Aeromonas, Clostridium sensu stricto, Lactobacillus, Halomonas, Ruminococcaceae, Shewanella, Sporolactobacillus, Romboutsia*, and *Bradyrhizobium* differed significantly between the H5N1-positive samples and the healthy control samples (*p* < 0.01, Mann-Whitney *U*-test).

### Decreased bacterial diversity of the fecal microbiota associated with H5N1-infection

An influence of avian influenza virus infection on gut microbial communities has been previously reported in mice model system (Dilantika et al., 2010; Ichinohe et al., 2011; Wang et al., 2014; Qin et al., 2015; Deriu et al., 2016). To further determine whether H5N1 infection altered the composition of the gut microbiota in migratory birds, we compared the diversity and richness indices between the two groups. The fecal microbiota of H5N1-infected individuals displayed greater overall diversity, with greater evenness and richness, as measured by the Shannon (*p* < 0.01; Figure 2B, Table 1) and Simpson diversity indices (*p* < 0.01; Figure 2B, Table 1).

**Table 1:** Comparison of phylotype coverage and diversity index at 97% similarity.

**Sample name**	**No. of reads**	**No. of OTUs**	**Chao 1 average**	**Observed species average**	**Shannon diversity index**	**Simpson diversity index**
H5N1-positive	54,598	361	367.75	307.30	2.29	0.56
Controls	48,176	688	677.95	596.60	3.51	0.67

In contrast to the diversity indices, however, there were significant differences in the richness indices (observed species and ACE) between the two groups (*p* < 0.01 and *p* < 0.05, Supplementary Figure 1, Table 1). The rarefaction curves showed that the total richness of the microbial community had been sampled completely. To better understand the differences in richness between the two groups, the overlap of the core microbial communities between the groups was illustrated using a Venn diagram. This analysis showed that only 596 of the 2,271 OTUs accounting for the total richness were common to all of the samples (Figure 2C). These data demonstrated that approximately 1,100 of the OTUs identified in the healthy-control samples were not detected in H5N1-positive samples.

**Figure 3:**
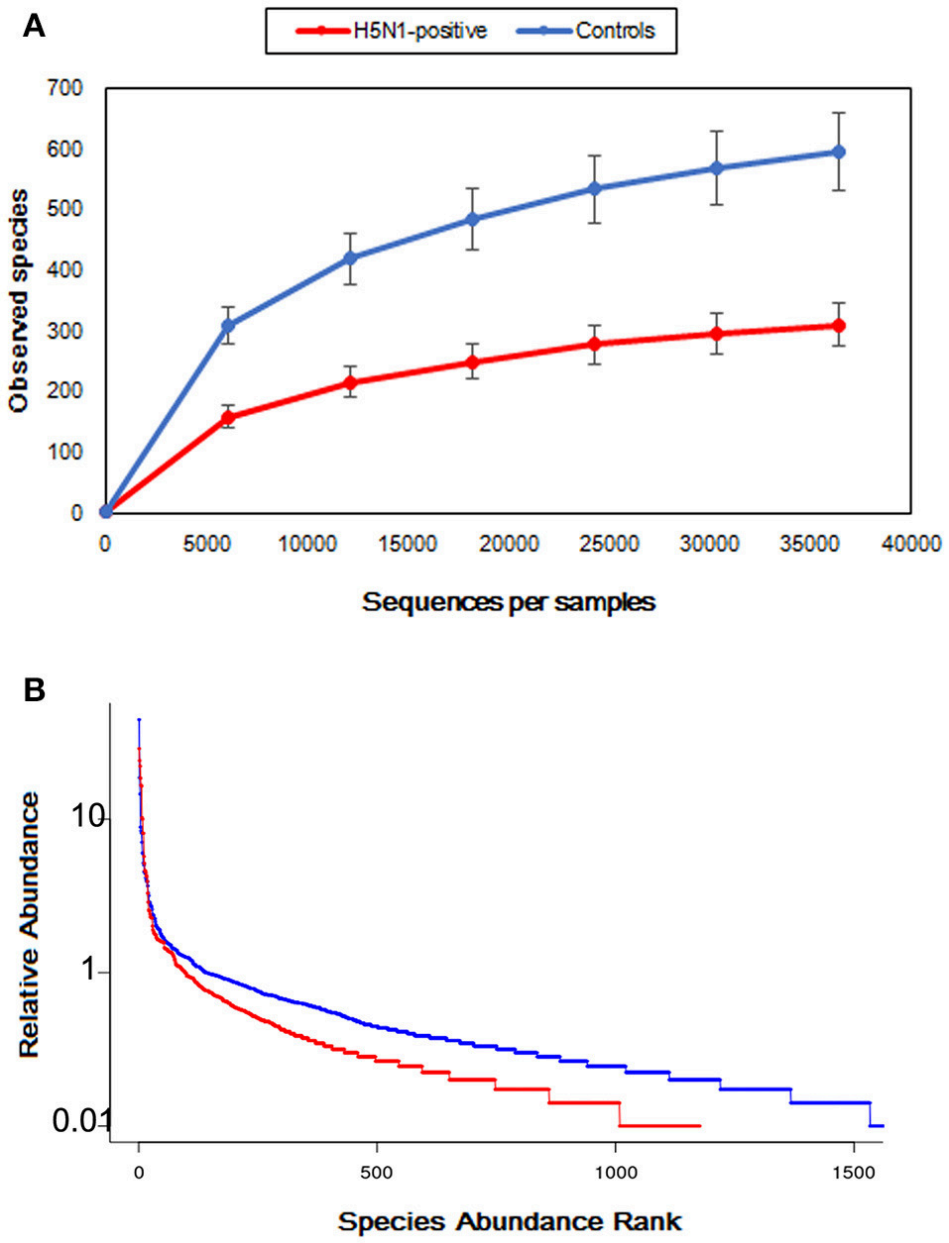
Comparison of the structures of the fecal microbiota of the healthy control, and H5N1-positive groups. **(A)** Structural comparison of the two groups. The vertical axis shows the number of OTUs that would be expected to be found after sampling the number of tags or sequences shown on the horizontal axis. **(B)** Rank abundance curve of the bacterial OTUs derived from the two groups.

Moreover, the rarefaction analysis curves also showed that the species richness of the fecal microbiota in swans with H5N1 virus infection tended to be lower than that in healthy controls (Figure 3A and Table 1). The rank abundance curves for the bacterial communities of the two groups indicated similar pattern (Figure 3B). The long tail of the rank abundance curve indicated that the majority of OTUs were present at low abundance in the fecal microbiota.

### H5N1 infection destabilizes the composition of the fecal microbiota

**Figure 4:**
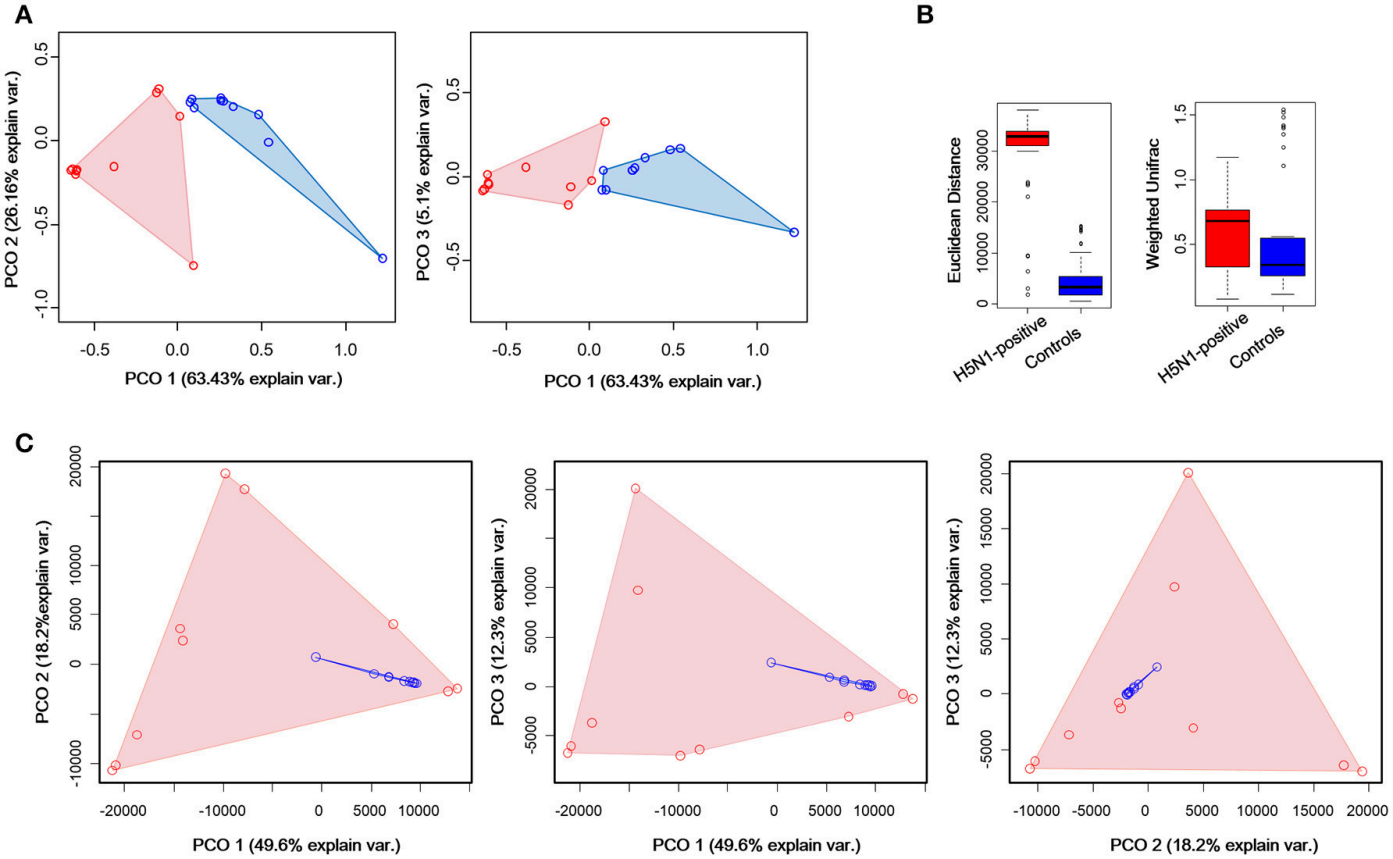
Greater compositional heterogeneity among H5N1-infected gut microbiomes than among uninfected controls. **(A)** The difference in the composition of the gut microbiota is illustrated by principal-coordinate plots of the weighted unifrac matrix among samples. All pairwise comparisons involved the first, second and third principal axes. The first, second and third principal axes explained most of the variance (63.43, 26.16, and 5.1%, respectively). Dots and surrounding areas correspond to gut bacteria communities from healthy controls (blue) and H5N1-infected individuals (red). **(B)** The relative abundance of bacterial phylotypes between the H5N1-infected samples (red bars) and the healthy control samples (blue bars). Asterisks indicate statistically significant differences (*p* < 0.01), and the error bars denote 95% confidence intervals for the mean values. **(C)** The difference in the composition of the gut microbiota is illustrated by principal-coordinate plots of the Euclidean distances among samples. All pairwise comparisons involved the first, second and third principal axes. The first, second and third principal axes explained most of the variance (58.3, 26.3, and 12.6%, respectively). Dots and surrounding areas correspond to gut bacteria communities from healthy controls (blue) and H5N1-infected individuals (red).

To measure the degree to which the fecal microbiota of the H5N1-infected individuals differed from that of the healthy controls, Principal Coordinates Analysis (PCoA) was conducted based on the Weighted Unifrac matrix and Euclidean distance matrix between the fecal samples. This analysis showed a strong difference in the microbiota of individuals with H5N1 infection compared to that of healthy controls. Despite significant inter-individual variation, the fecal microtiota of the two groups could be clearly separated using PCoA by weighted unifrac method (Figure 4A). To detect changes in the frequencies of dominant community constituents, we conducted PCoA based on Euclidean distances, and found that community composition of the H5N1-infected individuals differed significantly in composition from those of the fecal microbiota of the healthy controls (Figure 4C). Euclidean distance metrics and Weighted Unifrac metrics showed that in terms of changes in frequency, relative abundance and the presence or absence of bacterial phylotypes, the communities of the H5N1-infected individuals were more variable than those of the healthy controls (*p* < 0.01; Figure 4B). These results indicated that an increased level of β diversity was found in the fecal microbiota of the H5N1-infectious individuals.

As for the k-means clustering, optimal number of clusters for k-means was determined using the average silhouette width of all clusters (the distance metric defaults to euclidean) as criterion and K-means++ as initializer. *K* = 2 was chosen as the optimal number of clusters (Supplementary Figure 2). K-means clustering by Principal Component Analysis (PCA), showed that all the control samples and four H5N1-positive samples were clustered together while the other H5N1 samples were clustered together (Supplementary Figure 3).

### H5N1-associated alterations in fecal microbiota

**Figure 5:**
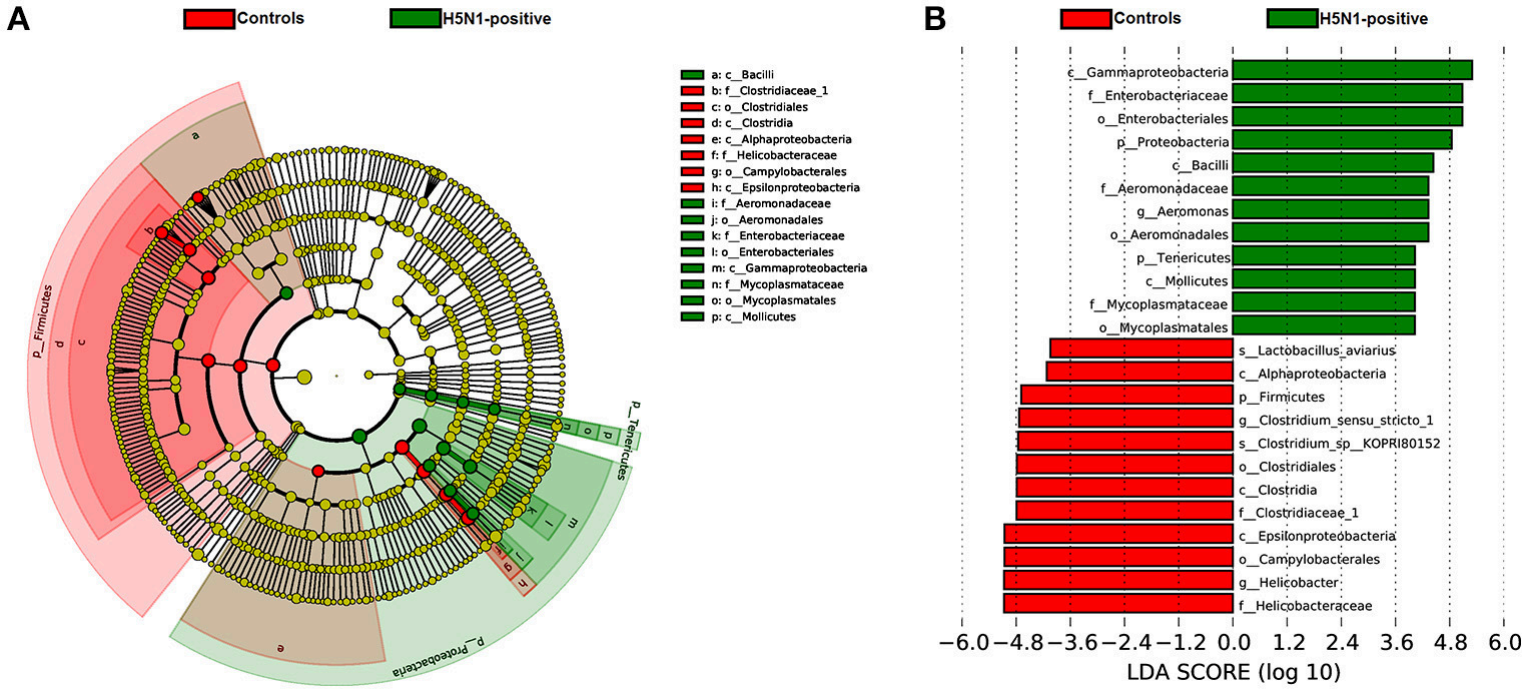
The taxa whose abundance differed between the H5N1-infected individuals and the healthy controls were identified by LEfSe. **(A)** Taxonomic cladogram obtained from LEfSe analysis of sequences (relative abundance ≥0.5%). Biomarker taxa are highlighted by colored circles and shaded areas (H5N1-positive samples are shown in green, and healthy control samples are shown in red). Each circle's diameter reflects the abundance of that taxa in the community. **(B)** The taxa whose abundance differed between the H5N1-infected samples (green bars) and the healthy control samples (red bars). The cutoff value of ≥2.0 used for the linear discriminant analysis (LDA) is shown.

To identify the specific bacterial taxa associated with H5N1 infection, we compared the fecal microbiota of healthy controls and H5N1-infected individuals using the linear discriminant analysis (LDA) effect size (LEfSe) method. A cladogram representative of the structure of the fecal microbiota and the predominant bacteria is shown in Figures 5A,B; the greatest differences (LDA > 2) in taxa between the H5N1-positive group and the healthy controls are displayed.

**Figure 6:**
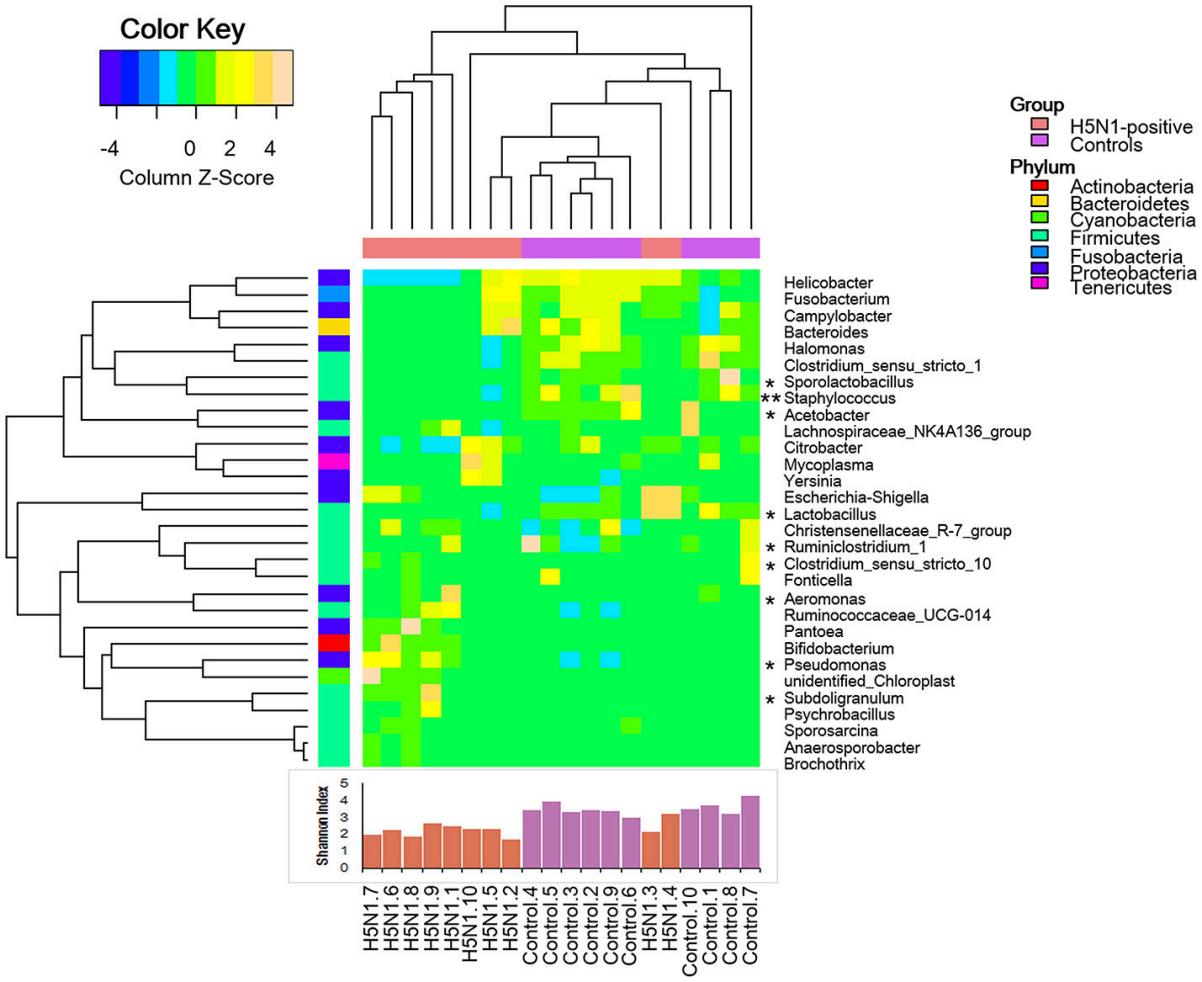
Heatmap illustrating genus-level differences between the H5N1-infected individuals and the healthy controls. The relative abundance of each genus is indicated by a Z score, and a gradient of color from blue to yellow indicates the abundance, ranging from low to high. The gut microbiota was found to have a distinctive bacterial composition, which was also significantly associated with the Shannon index. ^*^p < 0.05 and ^**^p < 0.01 by Mann-Whitney *U*-test.

Moreover, consistent with alpha diversity indices such as the Shannon index and with the beta diversity metrics such as PCoA, clustering analysis (the relative abundance of each genus is indicated by a Z score) of the top 30 genera highlighted differences in their distributions due to H5N1 infection. Clearly, the different composition in some genera of the fecal microbiota was associated with H5N1 infection (^*^p < 0.05 and ^**^p < 0.01 by Mann-Whitney *U*-test were shown in Figure 6).

## Discussion

Influenza is an infectious respiratory disease affecting many bird and mammal species (Laver and Webster, 1979). There are lots of researches focused on the role of antiviral immune against influenza virus infections (Iwasaki and Pillai, 2014; Jiang et al., 2016; Li et al., 2017; Liu et al., 2017; Zheng et al., 2017). Clinically, influenza in humans is often accompanied by gastroenteritis-like symptoms such as diarrhea. A few of studies reported that the intestinal microbial community were related to these clinical manifestations in the intestine during a lung viral influenza infection. Ichinohe et al. (2011) revealed the importance of commensal microbiota in regulating immunity in the respiratory mucosa through the proper activation of inflammasomes. Using an influenza mouse model, Deriu et al. (2016) found that influenza infection alters the intestinal microbial community through a mechanism dependent on type I interferons induced in the pulmonary tract. Qin et al. (2015) found that H7N9 viral infection have a significant effect on the microbiota community with decreased diversity and overgrowth of the bacteria such as *Escherichia coli* and *Enterococcus faecium*, and in the H7N9 patients *Clostridium* sp. *7 2 43FAA* and *Enterococcus faecium* were enriched. Wang et al. (2014) found that respiratory influenza infection induces intestinal immune injury by altering the composition of intestinal microbiota, and the increase of *E. coli* may be the primary cause for intestinal immune injury during influenza infection. Our study provided the first field study that the intestinal microbial community played an important role in migrate lung influenza infection.

In this outbreak, the novel H5N1 reassortants were found to be highly pathogenic to whooper swans. Except for the viral encephalitis and severe lung damage, necroticans enteritis were observed as well. And there was no H5N1 virus detected in the intestine (Bi et al., 2015). In this study, the high-throughput sequencing of 16S rRNA genes was applied to analyses, for the first time, the fecal microbiota community characterization of the HPAI H5N1 virus (WS01) infected migratory birds, whooper swans. We set out to determine whether whooper swans fecal microbiota composition vary in response to H5N1 virus lung infection. A core microbiota composed of 596 OTUs compromising over 26% of the total number of reads was common to both of the two groups (Figure 2C). Our data suggest that there is a statistical relation among respiratory H5N1 infection and shifts of fecal microbiota in whooper swans, including the fecal microbiota richness, diversity and composition.

In general, similar to mammals infected with influenza virus (such as H1N1-infected mice and H7N9-infected patients), the within sample diversity of gut microbial communities in avian was also found to decrease in infectious group (Dilantika et al., 2010; Wang et al., 2014; Qin et al., 2015). The perturbation of the gut microbiome was characterized by an increase in the dominance of the genus *Aeromonas*, while a reduction in the dominance of the genus *Lactobacillus* (*p* < 0.01). It has reported that, *Aeromonas* are opportunistic pathogen in environment (Gao et al., 2013), and *Lactobacillus* are gastrointestinal commensals which can display probiotic properties (Barton and Hughes, 1981; Verma et al., 2006).

Meanwhile, an accompanying decrease in microbial diversity (Shannon and Simpson index) was found in H5N1-infected individuals compared to the healthy controls (Figure 2B), similar with hepatitis B virus-induced chronic liver disease and *Clostridium difficile* infected children (Ling et al., 2014, 2015). While different from the result of α diversity analysis, the gut microbiota of the H5N1-infected individuals occupied a greatly expanded area of compositional space than the abundance of gut communities in the healthy individuals (Figure 3, Supplementary Figures 2, 3). This parallels an effect of SIV infection in chimpanzees and HIV infection in human (Lozupone et al., 2013; Moeller et al., 2013). These observations indicated that respiratory H5N1 virus infection in whooper swan could disturb the homeostasis of the fecal microbiota. Interestingly, our study showed that the pattern of α diversity (within samples) differed markedly from comparisons between samples in the same disease/health state (β diversity). For example, the H5N1 infectious individuals had the lower median α diversities, but higher β diversity (Figures 2–4). It mean that although each individual's fecal microbial community was ecologically less abundant, the organisms were very different in members of the group. Similar with that, *Rausch* et al. also identified the parallel alterations in diversity between Crohn disease patients and healthy controls (Rausch et al., 2011). Moreover, the Human Microbiome Project Consortium and Lozupone et al. also found the similar alteration in human microbiome of different habitats (Human Microbiome Project Consortium, 2012).

However, K-means clustering and Principal Component Analysis (PCA), showed that all the control samples and three H5N1-positive samples were clustered together (Supplementary Figure 3). The disease states may contribute to the differences in the microbiota (Lozupone et al., 2012; Moeller et al., 2013). PCA showed that opportunistic pathogen from genera *Pseudomonas* and *Pantoea* were main bacterium community that drive the cluster of H5N1 infectious individuals, and symbionts in genera *Mycoplasma moatsii* and *Halomonas* contributed to the cluster of healthy controls. It is reports that genus *Pseudomonas* infections could occur in the skin, blood and lungs, and some species of genus *Pantoea* could result in soft tissue or joint infections (Cruz et al., 2007; Smith et al., 2014; Halder et al., 2015).

Several limitations in the present study should be acknowledged. First, the present study has been limited to comparisons of fecal microbiota within infected vs. uninfected individuals, as a Class II species in China, and migratory bird, we cannot to track the full composition of the fecal microbiota within the same individuals before and after IAV infection. Second, without mechanism revealing between H5N1 infection and fecal microbiota change, our study only referred to the pattern of fecal microbiota characteristic after infection. In future studies using specific pathogen free (SPF) duck and chicken models, we will evaluate the effect of selected gut bacteria on H5N1 (whooper swan isloate, WS01) infection and further transmission, and analyze the host immune response (local and system) to further evaluate the interaction mechanism between the underlying fecal microbiota changes and H5N1 infection.

In summary, the novel findings of the present study demonstrated the statistical association among H5N1 infection and fecal microbiota compositional shifts in whooper swan. This study of H5N1-infected whooper swan may help elucidate the role of respiratory influenza virus infection in shaping the composition of fecal microbiota in migrates. Moreover, the changes that were observed in the fecal microbiota of the H5N1-infected swans provide new evidence that properties of the microbiota may serve as the risk of gut-linked disease in individuals with H5N1. Alternatively, opportunistic pathogens might be the source of the increase in unique phylotypes in the fecal microbiota of H5N1-infected individuals.

There are approximately 20,000 whooper swans wintering in China, and more than half of these wild birds winter in the Sanmenxia Reservoir Area along with various ducks from the East Asian/Australasian and Central Asian flyways (China Whooper Swan Research Center, 2016). These birds arrive in the Sanmenxia Reservoir Area during early October for wintering, and then fly back to Mongolia and Siberia for breeding next spring (Supplementary Figure 4, BirdLife International, 2016). The recent outbreak again highlighted the importance of effective surveillance and conservation strategies. Our study may provide the first evidence that properties of the fecal microbiota may serve as the risk of gut-linked disease in migrates with H5N1, and further aggravate the disease transmission. Our findings of H5N1 statistically associated alterations in fecal microbiota hinted that, in the future studies, the integrated solutions at culture etiological detection and new insights targeting the microbiota may better offer powerful epidemiological tools in order to mitigate the factors affecting H5N1 virus infection.

## Accession numbers

The sequencing reads generated from the whooper swan individuals described in this study have been deposited in the sequence read archive (SRA) at NCBI under the accession number SRP091349.

## Author contributions

HH and NZ designed the study. NZ and WS collected samples. NZ, HL, SL, and ML facilitated DNA sequencing. NZ and SW prepared the manuscript. HH and JL reviewed and edited the final version. All authors read and approved the final manuscript.

### Conflict of interest statement

The authors declare that the research was conducted in the absence of any commercial or financial relationships that could be construed as a potential conflict of interest. The handling Editor declared a shared affiliation, though no other collaboration, with one of the authors NZ.
