# Supplementary material for: Influence of Novel Highly Pathogenic Avian Influenza A (H5N1) Virus Infection on Migrating Whooper Swans Fecal Microbiota
Source: Front Cell Infect Microbiol. 2018 Feb 22;8:46. doi: 10.3389/fcimb.2018.00046 (PMC5827414; doi:10.3389/fcimb.2018.00046)
Supplement: Supplementary file 1 [file Presentation1.PDF]

Supplementary materials

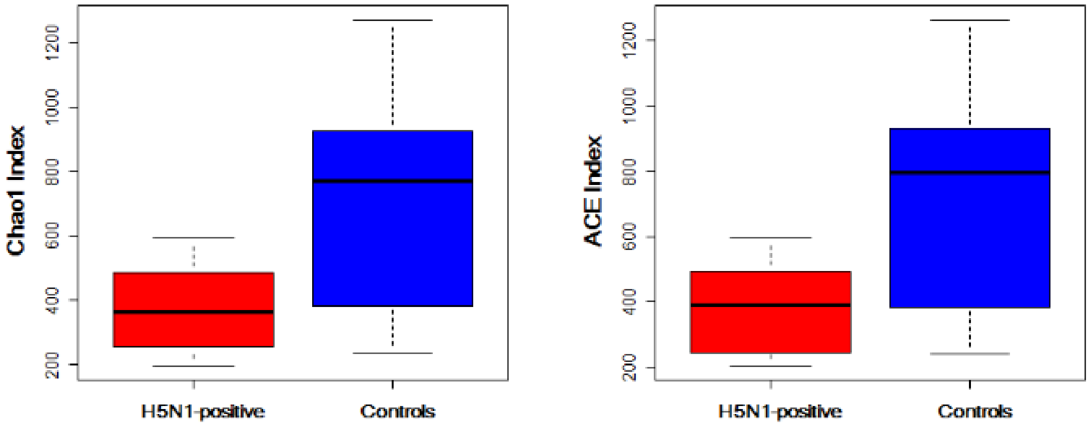

Supplementary Figure 1. Estimators of richness and diversity used to compare the gut microbiota of the H5N1-positive samples and the healthy control samples. The Chao 1 index (A) and ACE richness index (B) were calculated from the sequencing data using QIIME (Version 1.7.0) at 97% similarity to estimate the richness.

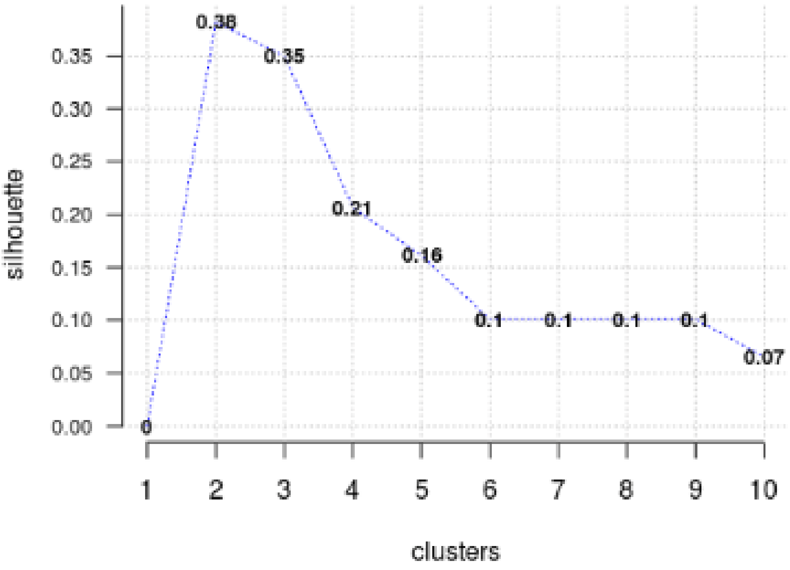

Supplementary Figure 2. Optimal number of clusters for k-means was determined using the average silhouette width of all clusters (the distance metric defaults to euclidean) as criterion and K-means++ as initializer.

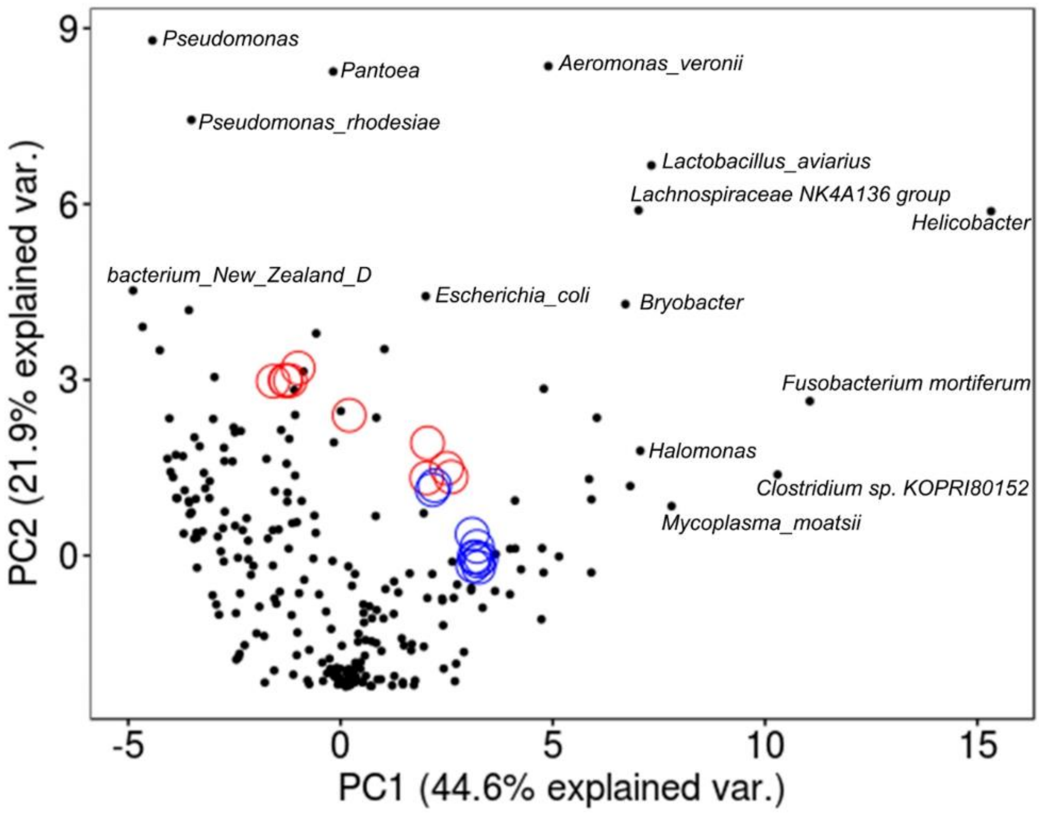

Supplementary Figure 3. Principal Component Analysis (PCA) was performed using log-transformed OTU matrix. The first and second principal component explain most of the variance (44.6% and 21.9%, respectively). Black dots respected the OTUs abundance. H5N1-positive samples were showed in red circles, and healthy controls were showed in blue circles. The OTUs in the upper left contributed to the cluster of the H5N1-positive samples, and those in the lower right contributed to the cluster of the healthy controls.

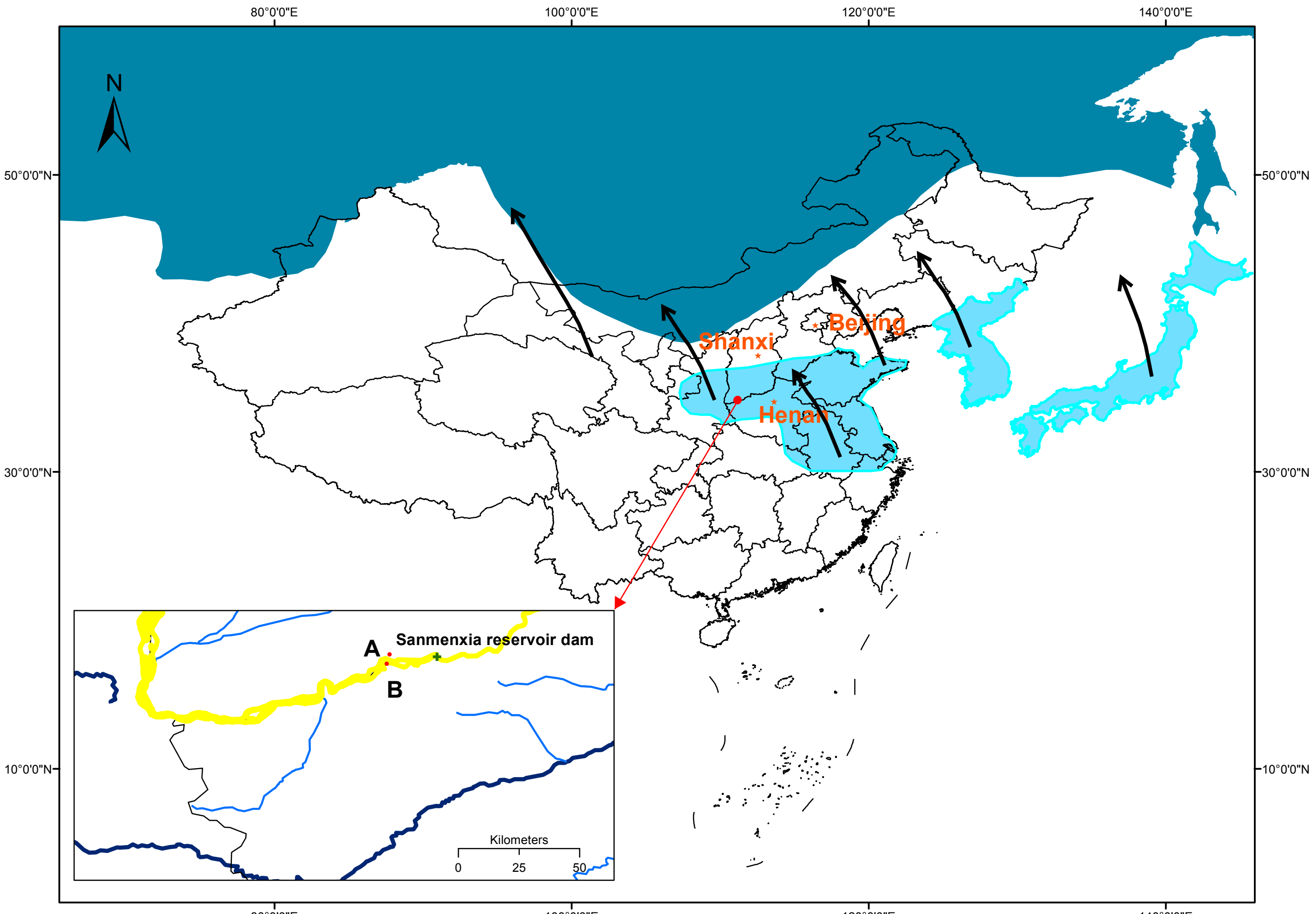
80°0'0"E
100°0'0"E
120°0'0"E
140°0'0"E
50°0'0"N
30°0'0"N
10°0'0"N
N
Beijing
Shanxi
Henan
A
B
Sanmenxia reservoir dam
Kilometers
0
25
50

Supplementary Figure 4. Outbreaks area from which avian influenza A (H5N1) viruses were isolated: Sanmenxia Yellow River Reservoir of China (red dot), a wintering center for whooper swan that congregate from Siberia. Dark blue indicates their breeding range. Light blue indicates wintering range in Asia. Symbols on inset maps represent two sampling locations: (A) indicates the wetland of the Yellow River in Pinglu County, north China's Shanxi Province; (B) indicates the Swan Lake National Urban Wetland Park of Sanmenxia city, China's Henan Province.
